# Examining the relation between emotional experiences and emotional expressions in competitive tennis matches

**DOI:** 10.3389/fpsyg.2023.1287316

**Published:** 2024-01-05

**Authors:** Julian Fritsch, Janis Fiedler, Antonis Hatzigeorgiadis, Darko Jekauc

**Affiliations:** ^1^Institute of Sports and Sports Science, Karlsruhe Institute of Technology, Karlsruhe, Germany; ^2^Department of Physical Education and Sport Science, University of Thessaly, Trikala, Greece

**Keywords:** emotion components, push effect, pull effect, non-verbal behavior, emotion regulation

## Abstract

**Introduction:**

Emotions consist of different components such as the emotional experience, physiological reactions, action tendencies, or emotional expressions. Assessing the relation of these components may help to better understand the role of emotions in sport competitions. Based on the component process model of emotions, the goal of the present study was to assess the relation between emotional experiences and emotional expressions.

**Methods:**

Twenty tennis players (7 female) with an average age of 23.10 (SD = 4.88) were taped during competitive tennis matches. Thereafter, in individual meetings, after having watched themselves on the footage at pre-selected points, the players had to indicate whether, immediately after the end of a rally, they had a positive or a negative emotional experience and, in case they had one, rate its intensity. Moreover, based on their observable behavior, the players were also asked to state whether they could recognize a positive or a negative emotional expression and, in case they recognized one, rate its intensity. The occurrence of emotional expressions was additionally rated by two external coders. Using multilevel models, separate analyses were conducted for positive emotions after won points and negative emotions after lost points.

**Results and discussion:**

For both positive and negative emotions, the results indicated a positive correlation between emotional experiences and emotional expressions. Moreover, the intensity of emotional experiences could predict the occurrence of an emotional expression rated by both the players or external coders. These insights into the relation between emotional experiences and emotional expressions may help players to more effectively regulate their emotions.

## 1 Introduction

The way tennis players appraise a situation can be associated with different observable reactions after winning or losing a point in a match. After losing a point, a player may shake the head and give out a loud shout, while the same player in another situation may show no visible reaction. The fact that emotions can be seen externally indicates that emotions can be viewed as both intrapersonal and social events (Tamminen and Bennett, 2017). The players’ reactions also point to the distinction between the subjective experience and emotional expressions as two emotion components (Mauss and Robinson, 2009). From a methodological perspective, observational study designs to assess emotional expressions allow studying emotions “online” during a real sport competition (Fritsch et al., 2018). This procedure offers a methodological advantage over the assessment of subjective experiences, which is typically conducted with questionnaires (Jones et al., 2005) or interview techniques (Martinent and Ferrand, 2009), where for practical reasons the assessment typically takes place either before or after the sports competition.

Studies using observational designs to investigate the role emotional expressions in sports allow to examine (a) what situational factors are associated with subsequent emotional expressions and (b) the extent to which emotional expressions are associated with subsequent indicators of sports performance (Fritsch et al., 2020). Regarding the former, research has demonstrated that the importance of a situation, as indicated by the type of competition (e.g., elimination vs. group stage), the timing within a match, or the point difference at the current score, can influence whether players exhibit emotional expressions following the outcome of a point in a competition (Moesch et al., 2015; Fritsch et al., 2020). These situational factors are indicative of appraisal processes that influence the quality and intensity of emotional responses (Jekauc et al., 2021). For example, winning a point at the end of the match is likely to be appraised as more important than winning a point at the beginning of a match, thus increasing the likelihood of an emotional expression.

Research on the effects of emotional expressions on sport performance is equivocal. While positive emotional expressions after scoring in a football penalty shootout were positively associated with winning the shootout (Moll et al., 2010), no effect of emotional expressions on the outcome of the subsequent point in table tennis has been observed (Fritsch et al., 2020). Moreover, in handball, it was shown that the expression of positive emotions after scoring a goal might enhance sports performance when the team is currently playing well, but that it can have a negative effect on sports performance when the team is currently playing poorly (Moesch et al., 2018). These findings indicate the potential of observational tools focusing on emotional expressions to contribute to a better understanding of the role of emotions in sports. However, a limitation of such purely observational study designs is that the underlying reason for the observable behavior is not clear (Fritsch et al., 2020). It remains to be investigated whether players’ emotional expressions correspond to their subjective experiences, which is the focus of the present study.

A theoretical model that addresses the different emotion components is the *component process model* by Scherer (1984), which has recently been applied in the sports context (Jekauc et al., 2021). The component process model, which belongs to the family of appraisal theories (e.g., Arnold, 1960; Lazarus, 1991), distinguishes between physiological processes, motivational changes, motor expressions, and the subjective emotional experience of an emotion (Scherer, 2004). Physiological processes refer to changes in the autonomic nervous system, while motivational changes are related to one’s action tendencies (e.g., approach vs. withdrawal). Motor expressions involve observable changes in an individual’s facial expressions, gestures, posture, or verbalizations. Finally, the subjective experience refers to the consciously available feeling of an emotion and is often considered as the most essential part that distinguishes an emotion from other psychological states (Scherer, 2009). The component process model postulates that, based on appraisal processes, the different emotion components are related to each other through a complex process of synchronization (Scherer, 1984).

An emotional experience, however, does not always correspond with an individual’s emotional expression (Fernández-Dols and Ruiz-Belda, 1995; Fritsch et al., 2022d). For example, players may feel bad because they have made a mistake, but they do not show this outwardly. It is assumed that the outward signs of an emotion, manifested through different body channels, are influenced by push and pull effects (Scherer and Bänziger, 2010). On the one hand, push effects represent internal processes reflecting a direct indication of the underlying emotional state. On the other hand, pull effects represent more deliberate strategies that are used to create a certain impression through one’s own behavior (Scherer and Bänziger, 2010). Both push and pull effects contribute to a person’s emotional expression, and they should be considered proportional rather than mutually exclusive (Sander and Scherer, 2009). Thus, in some situations, internal emotional processes strongly influence one’s emotional expression (e.g., during an affective outburst), while social communication intentions may drive the expression in other situations (e.g., smiling after a defeat).

From an applied perspective, considering the effects emotional expressions can have on sports performance (e.g., Moll et al., 2010; Moesch et al., 2018), insights into the relation between emotional experiences and emotional expressions may help athletes regulate their emotions more effectively (Jekauc et al., 2021). Particularly the interplay between push and pull effects seems to have implications for the relation between emotional experiences and emotional expressions in sport. With regards to push effects, a higher emotional experience makes it more likely that an emotional expression is visible (Scherer and Bänziger, 2010). This reasoning is in line with the results of a qualitative study conducted in table tennis, suggesting that the emotional experience must exceed a certain threshold for an emotional expression to be shown (Fritsch et al., 2022d). Regarding pull effects, given the interpersonal effects of one’s body language (Furley and Schweizer, 2020), it is not surprising that players use strategies to change their emotional expression to influence the psychological state of the opponent (Sève et al., 2007; Fritsch et al., 2022d).

### 1.1 The present study

In the present study, we distinguish between positive and negative emotions rather than specific emotions such as anxiety, happiness, and so on. This decision was based on research indicating that the valence dimension of emotions captures meaningful differences in all components of emotions (for a review see Mauss and Robinson, 2009). The valence dimension reflects an individual’s valuation of whether something is beneficial (in case of positive emotions) or harmful (in case of negative emotions) to the individual’s wellbeing and goals (Jekauc et al., 2021). Moreover, a distinction is made between the *occurrence* and the *intensity* of emotions. On the one hand, consistent with the concept of just noticeable difference (Stern and Johnson, 2010), the occurrence may represent the threshold at which a change in an expression is observed as emotional. On the other hand, the intensity of an emotion illustrates the magnitude of an emotion and is assumed to be determined by the proportion of valence and arousal (for a discussion of how arousal and intensity are related, see Reisenzein, 1994). The emotional intensity can range from zero, when there is no emotion, and thus also no occurrence of emotion, to a very high intensity (Moors, 2009).

The purpose of the present study was to investigate the relation between emotional experiences and emotional expressions in competitive tennis matches. In sports like tennis or table tennis, players almost exclusively report positive emotions after winning a point and negative emotions after losing a point (Lewis et al., 2017). These findings may indicate that positive and negative emotions represent different processes (Cacioppo et al., 1999). Therefore, all hypotheses were separated for positive emotions after winning a point and negative emotions after losing a point. Based on the component process model of emotions (Scherer, 1984), we hypothesized (1) that the intensity of emotional experiences and emotional expressions would show a positive correlation at the within-person level. Moreover, because a certain threshold of the intensity of the emotional experience had been reported as necessary for emotions to be shown externally (Fritsch et al., 2022d), we (2) hypothesized that the intensity of the emotional experience would predict the occurrence of emotional expressions at the within-person level.

## 2 Material and methods

### 2.1 Sampling and participants

Tennis was chosen because the break between the points offers many opportunities for players to experience and show emotions. Tennis players were contacted through tennis coaches, representatives of clubs, or friends. In total, the sample^1^ was comprised of 20 tennis players (7 female), with 14 players from Germany and six from Denmark. The players were on average 23.10 years old (SD = 4.88), had played tennis for an average of 14.70 years (SD = 5.58), and had taken part in tennis competitions for an average of 10.85 years (SD = 4.91). The current level of the players ranged from international (*n* = 4), to national (*n* = 6), to regional (*n* = 10). The players trained on average 3.25 (SD = 1.67) days or 8.35 (SD = 8.78) hours per week.

### 2.2 Procedure

**FIGURE 1:**
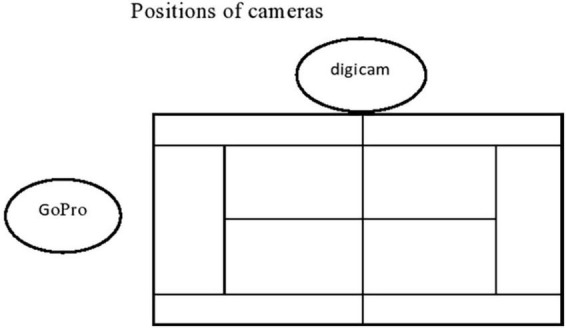
Positions of cameras. GoPro hung on the safety net behind the court and focused on the entire court. The digicam was on a tripod near the net and focused on the participating player.

The goal of the procedure was to measure emotional expressions and emotional experiences during real sports competitions. To that end, a naturalistic video-assisted procedure (e.g., Martinent and Ferrand, 2009) was employed during matches that were either part of an official tournament or of the regular season. Two cameras were positioned on the court as shown in Figure 1 (for a more detailed description see Supplementary File 1). To reduce the risk of self-presentational bias, the explicit focus on emotions was not mentioned to the players before the match. Instead, players were told that the study would investigate psychological processes during tennis matches in general. Consistent with the researcher’s observations, the players unanimously stated that due to the competitive nature of the match, their behavior was not influenced by the presence of the camera.

The first author of the study arranged individual meetings with the players. To ensure the accuracy of the players’ memories of their emotions, if feasible for the participants, meeting one day after the match was the preferred option. It was preferable to not hold the meeting on the same day as the match, because the preparation for each interview, including selecting the rallies and preparing the footage, took about 3 h. In addition, the players were often mentally and physically exhausted after the competition. One meeting took place on the same day of the competition, 17 meetings the day after the competition, and two meetings two days after the competition (*M*_*hours*_ = 22.65; SD = 9.23). The researcher prepared a preselection of 40 rallies to be assessed during the session (for the rationale of this preselection see Supplementary File 1). However, the emotions in these situations were subsequently rated by the players and external coders (see the section “2.3 Measures”). Thus, it was possible that the players or the external coders recognized an emotional expression that the researcher did not or vice versa. In the statistical analyses, only the ratings of the players and of the external coders were considered and not the ones of the researcher. For this reason, the researcher’s preselection of rallies had no direct influence on the statistical analyses.

At the beginning of each individual session, the researcher explained the difference between an emotional experience and an emotional expression. He explained that the emotional experience refers to the intrapersonal experience of an emotion. In comparison, an emotional expression would refer to the observable behavior based on the different body channels. All players confirmed that they understood the difference. Thereafter, the players were shown the 40 rallies chronologically, one after the other. Once the players had watched a rally from the footage of both cameras, they had to indicate whether they could recall it (from 1 = not at all to 7 = very clearly). Taking the context dependence of the memory into account (Smith and Vela, 2001), the outcomes of previous rallies and the current score were given as additional information. If the players reported their memory of the rally as “1,” no further questions concerning this rally were asked, and the next rally was shown. Moreover, if the players reported their memory of the rally as “2” or “3,” the researcher asked once more whether the player could actually recall the rally. This procedure intended to ensure that the players remembered the point at least to a certain degree. Only when the players were confident of their memory were they asked further questions about their emotional experiences and emotional expressions.

### 2.3 Measures

#### 2.3.1 Intensity of emotional experiences

The players were first asked whether they had experienced no emotion, a positive emotion, or a negative emotion after the rally. If the players reported experiencing a negative or a positive emotion, they were then asked to rate the intensity of the emotion (from 1 = very low to 7 = very high). If they reported experiencing no emotion, the intensity was scored as “0.” In light of the inter-individual differences in one’s emotionality (Reisenzein and Weber, 2009), players were asked to use their usual emotional experience as a reference point.

#### 2.3.2 Occurrence of emotional expressions

The players were asked to indicate from the footage whether they observed no emotional expression, a positive emotional expression, or a negative emotional expression after the rally. Specifically, players were told that a positive emotional expression meant that they had observed that something pleasant had happened based on their observable behavior. In contrast, a negative emotional expression meant that the players observed that something unpleasant had happened from their observable behavior.

#### 2.3.3 Intensity of emotional expressions

In case the players observed a positive or negative emotional expression, they also had to state the intensity of the emotional expression (from 1 = very low to 7 = very high). If they indicated that they had not observed any emotional expression, the intensity was counted as “0.” Again, due to the inter-individual differences in one’s emotionality (Reisenzein and Weber, 2009), players were asked to use their usual emotional expressions as a reference point.

#### 2.3.4 External ratings of occurrence of emotional expressions

The occurrence of emotional expressions was also rated by two external coders. These two coders were a male and a female sports science student and they were blind to the purpose of the study. The two coders were asked to indicate for every point included in the analyses, whether they observed no emotional expression, a positive emotional expression, or a negative emotional expression after the rally. Similarly to the players, the external coders were told that a positive emotional expression meant that they had observed something pleasant had happened. In contrast, a negative emotional expression meant that the coders observed that something unpleasant had happened. Notably, based on the finding that conclusions about an emotional state cannot be inferred from physical features alone (Kayyal et al., 2015), the outcome of the point was provided as relevant context information (for a similar procedure see Fritsch et al., 2020). For one player, for technical reasons, the footage could not be used for the external ratings. Thus, the 31 situations from this player were not used to assess the occurrence of emotional expressions by the external coders.

### 2.4 Statistical analysis

As there were multiple observations per player, we calculated multilevel models using RStudio (R Core Team, 2020). In the multilevel models, level 1 represented the variation due to the predictor in a match, which was nested within each player (level 2). A simulation study showed that multilevel analyses can be used to estimate regression coefficients for a number of only ten units at level 2 and five observations per unit at level 1 (Maas and Hox, 2005). Thus, although the sample in the present study was rather low (*n* = 20), the large number of observations per player allowed the use of multi-level analyses with a focus on the within-person associations by controlling for between-person effects (Hox, 2010). Because tennis players typically report positive emotions after winning a point and negative emotions after losing a point (Lewis et al., 2017), all analyses were conducted separately for positive emotions after won points and negative emotions after lost points.

Regarding the first hypothesis, we used the R package “correlation” (Makowski et al., 2020), which allowed us to calculate the correlations between the intensity of the emotional experiences and the intensity of the emotional expressions at the within-person level, taking into account between-person differences. Concerning the second hypothesis, assessing whether the intensity of emotional experiences would be positively associated with the occurrence of emotional expressions at the within-person level, we calculated multilevel logistic regressions within the R package “lme4” (Bates et al., 2015). A logistic regression was used, since the outcome variable “occurrence of emotional expressions” represents a categorical variable (“0” coded as no occurrence of an emotional expression and “1” coded as a “occurrence of positive/negative emotional expression”). The procedure of binary multilevel logistic regression was consistent with the steps recommended by Sommet and Morselli (2017). First, the predictor “intensity of emotional experience” was centered on the person-mean. Subsequently, we calculated a null model containing no predictor variable and calculated the intraclass correlation coefficient (ICC). The ICC was 0.29 for won points and 0.05 for lost points indicating the need for multi-level analyses. As a next step, we calculated a model with a random intercept and a fixed predictor (model 1) and compared it with a model with a random intercept and a random slope (model 2). To compare the different models, using ANOVA function within RStudio, the χ2-difference test was applied. Moreover, the akaike information criterion (AIC) of the two models could be compared with a lower AIC indicating a better model fit (Vrieze, 2012). Because there was no improvement of the models shown for the inclusion of random slopes, neither for won points (AIC model 1 = 339.04; AIC model 2 = 343.02; Δχ2 = 0.02, Δ*df* = 2, *p* = 0.99) nor for lost points (AIC model 1 = 328.31; AIC model 2 = 330.99; Δχ2 = 1.32, Δ*df* = 2, *p* = 0.52), the final models included a random intercept and a fixed slope.

For the external ratings of the occurrence of emotional expressions, we conducted two separate multi-level logistic regression analyses each for positive emotions after won points and for negative emotions after lost points: one analysis where the occurrence of an emotional expression was coded as “1” when both coders identified the corresponding emotional expression and one analysis where the occurrence of an emotional expression was coded as “1” when it was sufficient that the emotional expression was identified by at least one coder. Although for one model, including a random intercept and a random slope showed a slight improvement to the model including a random intercept and a fixed slope (ΔAIC = 5.60), for consistency reasons, all models were with a random intercept and a fixed slope (see the different fit indices in the Supplementary File 2).

## 3 Results

### 3.1 Memory of rallies

A total of 782 rallies (392 won and 390 lost) were shown to the tennis players. Due to sudden events, one player was unable to complete the session after seeing only 22 rallies. The players were able to recall a total of 692 rallies (88.49%), of which 335 were won (48.4%) and 357 lost (51.5%) points. The reported mean score of the players’ recall was 5.04 (SD = 2). For each player, emotional experiences and expressions were assessed in an average of 34.6 rallies (SD = 5.74). These rallies include an average of 16.75 (SD = 3.42) situations after won points and 17.85 (SD = 3.20) situations after lost points per player.

### 3.2 Descriptive statistics

The number of all situations is presented in Table 1, separated by won and lost points. The players reported only a few situations in which they had a negative emotional experience after a won point (*n* = 11/3.3%) and a positive emotional experience after a lost point (*n* = 4/1.1%). Furthermore, when players won points, they reported multiple situations (*n* = 121/36.1%), in which they had a positive emotional experience, but showed a neutral emotional expression. Similarly, when players lost points, they reported multiple situations (*n* = 77/21.6%), in which they had a negative emotional experience, but showed a neutral emotional expression. In contrast, when the players showed a positive or negative emotional expression after a point, there were only a few situations in which they did not report an emotional experience of the same valence (*n* = 3/0.1%, after won points; *n* = 3/0.1%, after lost points). In the following analyses, the focus was on the positive emotions after won points (*n* = 324) and negative emotions after lost points (*n* = 351). The intensity of both emotional experiences and emotional expressions across all situations and separated by won and lost points are shown in Table 2.

**TABLE 1:** Emotional experiences and emotional expressions after won and lost points.

After won points	Expression				After lost points	Expression			
**Experience**	**Neutral**	**Positive**	**Negative**	**Total**	**Experience**	**Neutral**	**Positive**	**Negative**	**Total**
Neutral	30	0	0	30	Neutral	33	1	1	35
Positive	121	173	0	294	Positive	2	2	0	4
Negative	5	3	3	11	Negative	77	1	240	318
Total	156	176	3	335	Total	112	4	241	357

The columns refer to the instances in which players showed an emotional expression, while the rows refer to the instance in which players reported an emotional experience. For example, after won points, the players reported in 294 situations a positive emotional experience. In 121 of these situations this experience was accompanied by a neutral emotional expression and in 173 of these situations this experience was accompanied by a positive emotional expression.

**TABLE 2:** Intensity of emotional experiences and emotional expressions.

	N situations	Intensity experience	Intensity expression
Total	692	3.85 (SD = 1.98)	2.34 (SD = 2.28)
Won points*	324	3.94 (SD = 1.99)	2.08 (SD = 2.31)
Lost points**	351	3.81 (SD = 1.99)	2.61 (SD = 2.24)

*Situations with negative emotions excluded (*n* = 11);

**Situations with positive emotions excluded (*n* = 6); In situations with a neutral emotional experience/expression, the intensity was considered “0.”

### 3.3 Multi-level correlation emotional experiences and emotional expressions

After won points, there was a significant within-person correlation between the intensity of positive emotional experiences and positive emotional expressions, *r*(322) = 0.58, CI = 0.50–0.65. Similarly, after lost points, there was a significant within-person correlation between the intensity of negative emotional experiences and negative emotional expressions, *r*(349) = 0.64, CI = 0.57–0.70.

### 3.4 Prediction of the occurrence of emotional expressions rated by the players

For won points, the ICC in the final model was 0.42. As shown in Table 3, the analysis revealed a significant within-person effect for the intensity of the emotional experience on the occurrence of an observed positive emotional expression (OR = 2.11; *p* < 0.01). For every unit increased in the intensity of the emotional experience, the chance of the occurrence of an observed positive emotional expression increased by the factor 2.11.

**TABLE 3:** Prediction of occurrences of emotional expressions.

	*b*	SE	OR_emotional_ _expression_	CI	*p*
**Won points**					
**Emotional expression rated by player**					
Intercept	0.09	0.37	1.08	0.52–2.27	0.84
Intensity of emotional experience	0.74	0.11	2.11	1.71–2.60	< 0.01
**Emotional expression rated by coders (criterion: expression by both coders identified)**					
Intercept	−1.15	0.28	0.32	0.18–0.55	< 0.01
Intensity of emotional experience	0.67	0.11	1.95	1.59–2.40	< 0.01
**Emotional expression rated by coders (criterion: expression at least by one coder identified)**					
Intercept	−0.06	0.27	0.94	0.56–1.59	0.816
Intensity of emotional experience	0.64	0.10	1.90	1.57–2.29	< 0.01
**Lost points**					
**Emotional expression rated by player**					
Intercept	1.08	0.21	2.93	1.96–4.39	< 0.01
Intensity of emotional experience	0.85	0.10	2.35	1.92–2.87	< 0.01
**Emotional expression rated by coders (criterion: expression by both coders identified)**					
Intercept	0.34	0.18	1.40	0.99–2.00	0.06
Intensity of emotional experience	0.52	0.08	1.68	1.44–1.97	< 0.01
**Emotional expression rated by coders (criterion: expression at least by one coder identified)**					
Intercept	1.64	0.21	5.13	3.37–7.82	< 0.01
Intensity of emotional experience	0.52	0.09	1.68	1.40–2.02	< 0.01

For lost points, the ICC in the final model was 0.11. The analysis showed a significant within-person effect for the intensity of the emotional experience on the occurrence of an observed negative emotional expression (OR = 2.35; *p* < 0.01). For every unit increased in the intensity of the emotional experience, the chance of the occurrence of an observed negative emotional expression increased by the factor 2.35.

### 3.5 Prediction of the occurrence of emotional expressions with external raters

In these analyses, 309 situations were coded after a won point and 334 situations were coded after a lost point by the two coders. For the 309 won points, in 95 situations (30.7%) both coders and in 155 situations (50.2%) at least one coder observed the occurrence of a positive emotional expression. As shown in Table 3, the analyses showed a significant within-person effect for the intensity of the emotional experience on the occurrence of an observed positive emotional expression for both criteria (when an expression had to be identified by both coders: OR = 1.95; *p* < 0.01/when an expression had to be identified by at least one coder: OR = 1.90; *p* < 0.01). For every unit increased in the intensity of the emotional experience, the chance of the occurrence of an observed positive emotional expression increased by the factor 1.95 or 1.90, respectively.

For the 334 lost points, in 191 situations (57.2%) both coders and in 267 situations (79.9%) at least one coder observed the occurrence of a negative emotional expression. As shown in Table 3, the analyses showed a significant within-person effect for the intensity of the emotional experience on the occurrence of an observed negative emotional expression for both criteria (when an expression had to be identified by both coders: OR = 1.68; *p* < 0.01/when an expression had to be identified by at least one coder: OR = 1.68; *p* < 0.01). For every unit increased in the intensity of the emotional experience, the chance of the occurrence of an observed negative emotional expression increased by the factor 1.68 in both cases.

## 4 Discussion

The goal of the present study was to examine the relation between emotional experiences and emotional expressions in competitive tennis matches. The analyses were conducted separately for positive emotions after won points and for negative emotions after lost points. In line with our first hypothesis, the results indicate a positive relation between the intensity of the emotion components. In addition, consistent with our second hypothesis, the intensity of the emotional experience could predict the occurrence of emotional expressions (both as rated by the players themselves and by external coders).

The finding that players primarily reported positive emotions after won points and negative emotions after lost points is consistent with previous studies (Lewis et al., 2017; Fritsch et al., 2020). This finding could be explained by appraisal processes that relate the outcome of a point to the individual’s assessment of whether an event is harmful or beneficial to the individual’s well-being and current goals (Jekauc et al., 2021). While winning a point is beneficial to the goal of winning a match and is therefore associated with positive emotions, losing a point is detrimental to the goal of winning a match and is therefore associated with negative emotions. At the same time, there were few instances of positive emotional experiences/expressions after a lost point and vice versa. These situations indicate that appraisal processes may not be exclusively influenced by the outcome of points, but possibly also by other factors, such as the way a point was played or the opponent’s behavior (Lewis et al., 2017).

In support of the first hypothesis, the strong correlation between the intensity of the emotional experiences and the intensity of the emotional expressions is in line with the postulations of the component process model of emotions (Scherer, 1984). According to the component process model of emotions, on the one hand, the motor expression component, together with the physiological and motivational components, is assumed to form the subjective emotional experience (Scherer, 1984). On the other hand, the subjective emotional experience also feeds back, leading to changes in the other components. In this regard, studies in sports also suggest that the external display of emotions is not only the result of an emotional experience but can also cultivate the emotional experience itself (e.g., Ronglan, 2007; Moesch and Apitzsch, 2012; Fritsch et al., 2022d). Thus, while the correlational design of the present study precludes drawing conclusions on the causality of the relation between the different components of emotions, much research in psychology points to the assumed reciprocal effects of the relation (e.g., Adelmann and Zajonc, 1989; Price et al., 2012).

Concerning the second hypothesis, the results have further shown that, for both positive and negative emotions, the intensity of the emotional experience could predict the occurrence of emotional expressions. In general, it is assumed that the higher the intensity of an emotion, the higher the correspondence between the different emotion components (Davidson, 1992; Mauss et al., 2005). While the cross-sectional design of the present study does not allow for causal interpretation, the findings of a qualitative study in table tennis suggest that the intensity of the emotional experience must exceed a certain threshold for an emotional expression to be shown (Fritsch et al., 2022d). This threshold may vary depending on players’ trait ability to regulate emotions (e.g., Laborde et al., 2011) as well as state-like psychological processes (e.g., ego depletion; Englert, 2017) during a competition. Regarding the latter, an in-game analysis assessing the mental state of players at several points during a sports competition could help to gain more insights into this relation.

Moreover, the results showed that the intensity of the emotional experience could predict the occurrence of emotional expressions when the latter was rated by external coders. While the consistent finding across the different ratings supports the robustness of this relation, the effects were smaller in the external ratings. Additionally, the study revealed that there were instances where the coders disagreed in their ratings of emotional expressions, highlighting the potential for individual differences in perception of observable emotional behavior (Barrett, 2012).

Based on the component process model of emotions, the visible behavior is influenced not only by the emotional experience (i.e., push effects), but also by more deliberate strategies used to create a certain impression of oneself (i.e., pull effects; Scherer and Bänziger, 2010). While the findings of the present study suggest that the intensity of players’ emotional experience contributes to the emotional expression, we did not ask the players for their emotion regulation strategies. However, given that it is easier for individuals to regulate how expressive they are than regulating the intensity of their emotional experience (Webb et al., 2012), this can help understand why players express emotions in one situation but not in another. In this regard, the finding that the intensity of emotional stimuli may influence the effectiveness of emotion regulation strategies (e.g., Sheppes et al., 2014; Rottweiler et al., 2018) points to the interactive effects of push and pull effects. In instances where an individual is experiencing intense emotions, deliberate regulation strategies may be more challenging to apply (Sander and Scherer, 2009). At the same time, proactive regulation strategies may also help to prevent a strong emotional experience and thus also reduce the likelihood of an emotional expression (Gross, 2015).

### 4.1 Applied and methodological implications

From an applied perspective, considering the various ways emotional expressions can positively or negatively influence sports performance (for a review on body language in sport see Furley and Schweizer, 2020), tennis players may benefit from various psychological skills that allow them to regulate their emotional responses according to the demands of the situation. For example, because the intensity of the emotional experience seems to increase the likelihood of an emotional expression, tennis players could use breathing techniques to regulate their psychophysiological response toward emotionally challenging situations during a competition (Laborde et al., 2022). Because emotionally challenging situations are often unavoidable in tennis, especially in matches where the stakes are high, mindfulness training can help tennis players deal with their emotional state in a more non-judgemental way (Noetel et al., 2019; Jekauc et al., 2022). This strategy may be particularly helpful in coping with adverse events during a tennis competition (e.g., a lost point), as tennis players tend to be more expressive after such events (Fritsch et al., 2022c,2023c). Additionally, considering that in certain situations tennis players may also benefit from showing emotional expressions, players may use strategies like imagery (Munroe et al., 2000) or self-talk (Fritsch et al., 2022a) to increase how emotional they are. At the same time, considering the reciprocal relation between the emotional experiences and emotional expressions (Adelmann and Zajonc, 1989; Price et al., 2012), tennis players may also use their body language for regulating their subjective experience. This strategy can also be useful when playing doubles tennis, as emotions can transfer between players (for a review on emotional convergence in sport see Fritsch et al., 2023b).

The findings of the present study have further methodological implications for the assessment of emotions in sports. It has been argued that the measurement of emotional expressions allows the study of emotions during real sports competitions and therefore has a high ecological validity (Fritsch et al., 2018). Since in almost all cases, in which the players reported expressing an emotion, they also reported experiencing an emotion with the same valence, studies with a purely observational design seem to measure “real” emotional experiences (Moll et al., 2010; Moesch et al., 2015; Fritsch et al., 2020). However, the finding that many experienced emotions are not externally displayed underscores the risk of exclusively relying on observational designs, which could miss many experienced emotions, particularly those with lower intensity. Thus, to gain a deeper understanding of the dynamic and fluctuating affective processes during a sports competition, the use of multiple methods (i.e., observational methods, self-report, physiological measures) may help capture the different emotion components and their interactions (Jekauc et al., 2021).

### 4.2 Limitations and future directions

The fact that players often reported experiencing a positive or negative emotion even though they did not show an emotional expression in these situations suggests that, in principle, a distinction could be made between emotional experiences and emotional expressions. Nevertheless, a limitation of the current study is that the players’ memory of their emotional experience may have been biased by seeing themselves in the footage. Moreover, the assessed rallies were pre-selected by the researcher. The intention behind this procedure was to include diverse situations in the analyses. Nevertheless, this pre-selection does not allow for any conclusions to be drawn about the typical prevalence of emotions during tennis matches. Although multiple situations were included for each player, the sample was limited to a small number of players and it leaves the question open as to whether the findings can be generalized to other tennis players or also to players of other sports with a similar structure (e.g., table tennis, badminton, volleyball). For example, in contrast to tennis, it has been shown that in a team sport such as volleyball, positive emotional expressions are easier to recognize than negative ones (Fritsch et al., 2023a). Another limitation is that this study focused on the obvious changes in individuals’ behaviors (e.g., gestures, postures, verbalizations). However, less obvious changes, reflecting underlying emotional motivations, are also relevant to sports performance, for example by altering reaction times (Bishop et al., 2009) or power output (Woodman et al., 2009). In the same vein, the present study relied solely on the subjective perception to rate emotional expressions, and an alternative approach may involve the use of coding systems that focus on specific physical features to assess emotional expressions (e.g., Moesch et al., 2015; Furley and Roth, 2021).

Given the complex nature of emotions, there are various directions for future research that could contribute to a better understanding of their role in sports. While the present study examined the relation between emotional experiences and expressions, one could also focus on the physiological processes that accompany emotions (e.g., Brimmell et al., 2018; Zur et al., 2019). Moreover, the current study focused on within-person associations and future research with larger sample sizes could explore between-person differences in emotional expressions and experiences (Reisenzein and Weber, 2009). Additionally, recent technological advances offer opportunities for analyzing vast amounts of data from real-time sports competitions (e.g., Hetland et al., 2018), which could further increase our understanding of emotions in sports. Finally, future research could also explore the differences in emotional expressions and experiences based on specific emotions such as anger, shame, and pride.

## 5 Conclusion

Investigating the interplay between the different emotion components can contribute to a better understanding of the role of emotions in sports. For this reason, the present study focused on the relation between emotional experiences and emotional expressions. The results revealed a relatively high correlation between these two components. Moreover, the study also found that the intensity of emotional experiences was a predictor of the occurrence of emotional expressions as rated by players themselves and external coders. With relevance to the assessment of emotions, these results suggest that when an emotional expression is observed, it is likely that an emotional experience of the same valence occurred. However, the use of such purely observational tools might overlook emotional experiences with low intensity. Therefore, we encourage future studies to assess multiple components of emotions to gain a more comprehensive understanding of their role in sports.

## Data availability statement

The raw data supporting the conclusions of this article will be made available by the authors, without undue reservation.

## Ethics statement

The studies involving humans were approved by the University of Thessaly, Department of Physical Education and Sport Science. The studies were conducted in accordance with the local legislation and institutional requirements. Written informed consent for participation in this study was provided by the participants.

## Author contributions

JuF: Conceptualization, Formal analysis, Investigation, Methodology, Writing – original draft. JaF: Formal analysis, Methodology, Writing – review & editing. AH: Conceptualization, Investigation, Supervision, Writing – review & editing. DJ: Conceptualization, Methodology, Supervision, Writing – review & editing.

## Footnotes

1 The current sample was also used by Fritsch et al. (2022b). The previous study focused on the relation between self-talk and emotions, while the present study focuses on the relation between emotional experiences and emotional expressions. Moreover, in the previous study, there were no external rating for the occurrences of emotional expressions. For this reason the rationales, aims, and results of each study are fundamentally different.

## References

[B1] Adelmann P. K. Zajonc R. B. (1989). Facial efference and the experience of emotion. *Annu. Rev. Psychol.* 40 249–280. 10.1146/annurev.ps.40.020189.001341 2648977

[B2] Arnold M. B. (1960). *Emotion and Personality. Psychological Aspects*, Vol. I. New York, NY: Columbia University Press.

[B3] Barrett L. F. (2012). Emotions are real. *Emotion* 12 413–429. 10.1037/a0027555 22642358

[B4] Bates D. Mächler M. Bolker B. Walker S. (2015). Fitting linear mixed-effects models using lme4. *J. Stat. Softw.* 67 1–48. 10.18637/jss.v067.i01

[B5] Bishop D. T. Karageorghis C. I. Kinrade N. P. (2009). Effects of musically-induced emotions on choice reaction time performance. *Sport Psychol.* 23 59–76. 10.1123/tsp.23.1.59

[B6] Brimmell J. Parker J. K. Furley P. Moore L. J. (2018). Nonverbal behavior accompanying challenge and threat states under pressure. *Psychol. Sport Exerc.* 39 90–94. 10.1016/j.psychsport.2018.08.003

[B7] Cacioppo J. T. Gardner W. L. Berntson G. G. (1999). The affect system has parallel and integrative processing components: Form follows function. *J. Pers. Soc. Psychol.* 76 839–855.

[B8] Davidson R. J. (1992). Prolegomenon to the structure of emotion: Gleanings from neuropsychology. *Cognit. Emot.* 6 245–268. 10.1080/02699939208411071

[B9] Englert C. (2017). Ego depletion in sports: Highlighting the importance of self-control strength for high-level sport performance. *Curr. Opinion Psychol.* 16 1–5. 10.1016/j.copsyc.2017.02.028 28813329

[B10] Fernández-Dols J.-M. Ruiz-Belda M.-A. (1995). Are smiles a sign of happiness? Gold medal winners at the Olympic Games. *J. Pers. Soc. Psychol.* 69 1113–1119. 10.1037/0022-3514.69.6.1113

[B11] Fritsch J. Ebert S. Jekauc D. (2023a). The recognition of affective states associated with players’ non-verbal behavior in volleyball. *Psychol. Sport Exerc.* 64:102329. 10.1016/j.psychsport.2022.102329 37665814

[B12] Fritsch J. Elbe A.-M. Hatzigeorgiadis A. (2018). Ein Plädoyer für eine verstärkte Berücksichtigung der Verhaltenskomponente in der sportpsychologischen Emotionsforschung [A call for an increased consideration of the behavioral component of emotion in sport psychology]. *Z. Sportpsychol.* 25 79–88. 10.1026/1612-5010/a000220

[B13] Fritsch J. Feil K. Jekauc D. Latinjak A. T. Hatzigeorgiadis A. (2022a). The relationship between self-talk and affective processes in sports: A scoping review. *Int. Rev. Sport Exerc. Psychol.* 10.1080/1750984X.2021.2021543 [Epub ahead of print].

[B14] Fritsch J. Finne E. Jekauc D. Zerdila D. Elbe A.-M. Hatzigeorgiadis A. (2020). Antecedents and consequences of outward emotional reactions in table tennis. *Front. Psychol.* 11:578159. 10.3389/fpsyg.2020.578159 33041951 PMC7522351

[B15] Fritsch J. Jekauc D. Elsborg P. Latinjak A. T. Reichert M. Hatzigeorgiadis A. (2022b). Self-talk and emotions in tennis players during competitive matches. *J. Appl. Sport Psychol.* 34 518–538. 10.1080/10413200.2020.1821406

[B16] Fritsch J. Leis O. Wohlfarth D. Triller N. Jekauc D. (2023b). The role of emotional convergence in sport: A scoping review. *Sport Exerc. Perf. Psychol.* 10.1037/spy0000330 [Epub ahead of print].

[B17] Fritsch J. Preine L. Jekauc D. (2022c). The examination of factors influencing the recognition of affective states associated with tennis players’ non-verbal behavior. *Psychol. Sport Exerc.* 64:102206. 10.1016/j.psychsport.2022.102206 37665814

[B18] Fritsch J. Redlich D. Latinjak A. Hatzigeorgiadis A. (2022d). The behavioural component of emotions: Exploring outward emotional reactions in table tennis. *Int. J. Sport Exerc. Psychol.* 20 397–415. 10.1080/1612197X.2021.1877324

[B19] Fritsch J. Seiler K. Wagner M. Englert C. Jekauc D. (2023c). Can you tell who scores? An assessment of the recognition of affective states based on the nonverbal behavior of amateur tennis players in competitive matches. *J. Sport Exerc. Psychol.* 45 138–147. 10.1123/jsep.2022-0182 37185449

[B20] Furley P. Roth A. (2021). Coding body language in sports: The nonverbal behavior coding system for soccer penalties. *J. Sport Exerc. Psychol.* 43 140–154. 10.1123/jsep.2020-0066 33730693

[B21] Furley P. Schweizer G. (2020). “Body language in sport,” in *Handbook of Sport Psychology*, eds Tenenbaum G. Eklund R. C. (Hoboken, NJ: Wiley), 1201–1219. 10.1002/9781119568124.ch59

[B22] Gross J. J. (2015). Emotion regulation: Current status and future prospects. *Psychol. Inq.* 26 1–26. 10.1080/1047840X.2014.940781

[B23] Hetland A. Vittersø J. Oscar Bø Wie S. Kjelstrup E. Mittner M. Dahl T. I (2018). Skiing and thinking about It: Moment-to-moment and retrospective analysis of emotions in an extreme port. *Front. Psychol.* 9:971. 10.3389/fpsyg.2018.00971 29973894 PMC6019491

[B24] Hox J. J. (2010). *Quantitative Methodology Series. Multilevel Analysis: Techniques and Applications*, 2nd Edn. Oxford, NY: Taylor & Francis Group.

[B25] Jekauc D. Fritsch J. Latinjak A. T. (2021). Toward a theory of emotions in competitive sports. *Front. Psychol.* 12:790423. 10.3389/fpsyg.2021.790423 34975686 PMC8716387

[B26] Jekauc D. Mülberger L. Weyland S. (2022). *Achtsamkeitstraining im Sport [Mindfulness training in sport].* Berlin: Springer.

[B27] Jones M. V. Lane A. M. Bray S. R. Uphill M. Catlin J. (2005). Development and validation of the sport emotion questionnaire. *J. Sport Exerc. Psychol.* 27 407–431. 10.1123/jsep.27.4.407

[B28] Kayyal M. Widen S. Russell J. A. (2015). Context is more powerful than we think: Contextual cues override facial cues even for valence. *Emotion* 15 287–291. 10.1037/emo0000032 25706831

[B29] Laborde S. Allen M. S. Borges U. Iskra M. Zammit N. You M. (2022). Psychophysiological effects of slow-paced breathing at six cycles per minute with or without heart rate variability biofeedback. *Psychophysiology* 59:e13952. 10.1111/psyp.13952 34633670

[B30] Laborde S. Brüll A. Weber J. Anders L. S. (2011). Trait emotional intelligence in sports: A protective role against stress through heart rate variability? *Pers. Individ. Differ.* 51 23–27. 10.1016/j.paid.2011.03.003

[B31] Lazarus R. (1991). *Emotion and Adaptation.* Oxford: Oxford University Press.

[B32] Lewis F. R. Knight C. J. Mellalieu S. D. (2017). Emotional experiences in youth tennis. *Psychol. Sport Exerc.* 29 69–83. 10.1016/j.psychsport.2016.12.003

[B33] Maas C. J. M. Hox J. J. (2005). Sufficient sample sizes for multilevel modeling. methodology. *Eur. J. Res. Methods Behav. Soc. Sci.* 1 86–92. 10.1027/1614-2241.1.3.86

[B34] Makowski D. Ben-Shachar M. S. Patil I. Lüdecke D. (2020). Methods and algorithms for correlation analysis in R. *J. Open Sour. Softw.* 5:2306. 10.21105/joss.02306

[B35] Martinent G. Ferrand C. (2009). A naturalistic study of the directional interpretation process of discrete emotions during high-stakes table tennis matches. *J. Sport Exerc. Psychol.* 31 318–336. 19798996 10.1123/jsep.31.3.318

[B36] Mauss I. Levenson R. McCarter L. Wilhelm F. Gross J. (2005). The tie that binds? Coherence among emotion experience, behavior, and physiology. *Emotion* 5 175–190. 10.1037/1528-3542.5.2.175 15982083

[B37] Mauss I. Robinson M. (2009). Measures of emotion: A review. *Cognit. Emot.* 23 209–237. 10.1080/02699930802204677 19809584 PMC2756702

[B38] Moesch K. Apitzsch E. (2012). How do coaches experience psychological momentum? A qualitative study of female elite handball teams. *Sport Psychol.* 26 435–453. 10.1123/tsp.26.3.435

[B39] Moesch K. Kenttä G. Bäckström M. Mattsson C. M. (2015). Exploring nonverbal behaviors in elite handball: How and when do players celebrate? *J. Appl. Sport Psychol.* 27 94–109. 10.1080/10413200.2014.953231

[B40] Moesch K. Kenttä G. Bäckström M. Mattsson C. M. (2018). Nonverbal post-shot celebrations and their relationship with performance in elite handball. *Int. J. Sport Exerc. Psychol.* 16 235–249. 10.1080/1612197X.2016.1216148

[B41] Moll T. Jordet G. Pepping G. J. (2010). Emotional contagion in soccer penalty shootouts: Celebration of individual success is associated with ultimate team success. *J. Sports Sci.* 28 983–992. 10.1080/02640414.2010.484068 20544488

[B42] Moors A. (2009). Theories of emotion causation: A review. *Cognit. Emot.* 23 625–662. 10.1080/02699930802645739

[B43] Munroe K. J. Giacobbi P. R. Hall C. Weinberg R. (2000). The four Ws of imagery use: Where, when, why, and what. *Sport Psychol.* 14 119–137.

[B44] Noetel M. Ciarrochi J. Van Zanden B. Lonsdale C. (2019). Mindfulness and acceptance approaches to sporting performance enhancement: A systematic review. *Int. Rev. Sport Exerc. Psychol.* 12 139–175. 10.1080/1750984X.2017.1387803

[B45] Price T. F. Peterson C. K. Harmon-Jones E. (2012). The emotive neuroscience of embodiment. *Motivat. Emot.* 36 27–37. 10.1007/s11031-011-9258-1

[B46] R Core Team (2020). *R: A Language and Environment for Statistical Computing.* Vienna: R foundation for statistical computing.

[B47] Reisenzein R. (1994). Pleasure-arousal theory and the intensity of emotions. *J. Pers. Soc. Psychol.* 67 525–539. 10.1037/0022-3514.67.3.525

[B48] Reisenzein R. Weber H. (2009). “Personality and emotion,” in *The Cambridge Handbook of Personality Psychology*, eds Corr P. J. Matthews G. (Cambridge, MA: Cambridge University Press), 54–71. 10.1017/CBO9780511596544.007

[B49] Ronglan L. T. (2007). Building and communicating collective efficacy: A season-long in-depth study of an elite sport team. *Sport Psychol.* 21 78–93. 10.1123/tsp.21.1.78

[B50] Rottweiler A.-L. Taxer J. L. Nett U. E. (2018). Context matters in the effectiveness of emotion regulation strategies. *AERA Open* 4 1–13. 10.1177/2332858418778849

[B51] Sander D. Scherer K. R. (2009). *The Oxford Companion to Emotion and the Affective Sciences.* Oxford: Oxford University Press.

[B52] Scherer K. R. (1984). “On the nature and function of emotion: A component process approach,” in *Approaches to Emotion*, eds Scherer K. R. Ekman P. (Mahwah: Lawrence Erlbaum Associates, Inc), 293–317.

[B53] Scherer K. R. (2004). “Feelings integrate the central representation of appraisal-driven response organization in motion,” in *Feelings and Emotions: The Amsterdam Symposium*, eds Manstead A. Frijda N. Fischer A. (Cambridge: Cambridge University Press), 136–157. 10.1017/CBO9780511806582.009

[B54] Scherer K. R. (2009). The dynamic architecture of emotion: Evidence for the component process model. *Cognit. Emot.* 23 1307–1351. 10.1080/02699930902928969

[B55] Scherer K. R. Bänziger T. (2010). “On the use of actor portrayals in research on emotional expression,” in *Blueprint for Affective Computing: A Sourcebook*, eds Scherer K. R. Bänziger T. Roesch E. B. (Oxford: Oxford University Press), 166–177.

[B56] Sève C. Ria L. Poizat G. Saury J. Durand M. (2007). Performance-induced emotions experienced during high-stakes table tennis matches. *Psychol. Sport Exerc.* 8 25–46. 10.1016/j.psychsport.2006.01.004

[B57] Sheppes G. Scheibe S. Suri G. Radu P. Blechert J. Gross J. J. (2014). Emotion regulation choice: A conceptual framework and supporting evidence. *J. Exp. Psychol.* 143 163–181. 10.1037/a0030831 23163767

[B58] Smith S. M. Vela E. (2001). Environmental context-dependent memory: A review and meta-analysis. *Psychon. Bull. Rev.* 8 203–220. 10.3758/bf03196157 11495110

[B59] Sommet N. Morselli D. (2017). Keep calm and learn multilevel logistic modeling: A simplified three-step procedure using Stata, R, Mplus, and SPSS. *Int. Rev. Soc. Psychol.* 30 203–218. 10.5334/irsp.90

[B60] Stern M. K. Johnson J. H. (2010). “Just noticeable difference,” in *The Corsini Encyclopedia of Psychology*, eds Weiner I. B. Craighead W. E. (Hoboken, NJ: John Wiley & Sons, Inc), 803–804.

[B61] Tamminen K. A. Bennett E. V. (2017). No emotion is an island: An overview of theoretical perspectives and narrative research on emotions in sport and physical activity. *Qual. Res. Sport Exerc. Health* 9 183–199. 10.1080/2159676X.2016.1254109

[B62] Vrieze S. I. (2012). Model selection and psychological theory: A discussion of the differences between the Akaike information criterion (AIC) and the Bayesian information criterion (BIC). *Psychol. Methods* 17 228–243. 10.1037/a0027127 22309957 PMC3366160

[B63] Webb T. L. Miles E. Sheeran P. (2012). Dealing with feeling: A meta-analysis of the effectiveness of strategies derived from the process model of emotion regulation. *Psychol. Bull.* 138 775–808. 10.1037/a0027600 22582737

[B64] Woodman T. Davis P. A. Hardy L. Callow N. Glasscock I. Yuill-Proctor J. (2009). Emotions and sport performance: An exploration of happiness, hope, and anger. *J. Sport Exerc. Psychol.* 31 169–188. 10.1123/jsep.31.2.169 19454770

[B65] Zur I. Cooke A. Woodman T. Neil R. Udewitz R. (2019). Don’t make me angry! A psychophysiological examination of the anger–performance relationship in intermediate and elite fencers. *J. Appl. Sport Psychol.* 31 285–302. 10.1080/10413200.2018.1464079

