# Supplementary material for: Examining the relation between emotional experiences and emotional expressions in competitive tennis matches
Source: Front Psychol. 2024 Jan 5;14:1287316. doi: 10.3389/fpsyg.2023.1287316 (PMC10799558; doi:10.3389/fpsyg.2023.1287316)
Supplement: Supplementary file 1 [file Data_Sheet_1.docx]

**Rationale for camera positions**

The first camera, a GoPro Action Camera Hero, was placed behind the court in order to record the entire court, including the movement of both players and the trajectories of the ball. The footage from this camera was used to stimulate the players’ memories of specific rallies after the match. The second camera, a digicam, was near to the net and directed toward the player who participated in the study. Each time the players changed sides during the match, the lens of the camera was repositioned towards the court side of the participating player. The recordings from the second camera were supposed to aid the players’ memory as well as to capture their emotional expressions.

**Rationale for preselection of points**

The goal of the preselection was to include a sample of heterogeneous emotional responses. For this reason, based on his subjective perception, the researcher tried to select (a) ten rallies in which the players won a point and remained neutral, (b) ten rallies in which the players won a point and showed a positive emotional expression, (c) ten rallies in which the players lost a point and remained neutral, and (d) ten rallies in which the players lost a point and showed a negative emotional expression. For some players, the researcher could not identify ten rallies with a negative or a positive emotional expression. In these cases, in order to obtain 40 rallies for each player, the researcher chose rallies with the same outcome as substitutes (e.g., a won point where the players remained neutral instead of showing a positive emotional expression).
