# Supplementary material for: Examining the relation between emotional experiences and emotional expressions in competitive tennis matches
Source: Front Psychol. 2024 Jan 5;14:1287316. doi: 10.3389/fpsyg.2023.1287316 (PMC10799558; doi:10.3389/fpsyg.2023.1287316)
Supplement: Supplementary file 2 [file Data_Sheet_2.docx]

**Model fits for analysis with codings of external raters**

Won points

External rating with the criterion that the positive expression had to be identified by both coders:

AIC of model with random intercept and fixed slope: 319.39

AIC of model with random intercept and random slope: 323.39

External rating with the criterion that the positive expression had to be identified by at least one coder:

AIC of model with random intercept and fixed slope: 361.13

AIC of model with random intercept and random slope: 355.53

Lost points

External rating with the criterion that the positive expression had to be identified by both coders:

AIC of model with random intercept and fixed slope: 404.02

AIC of model with random intercept and random slope: 405.29

External rating with the criterion that the positive expression had to be identified by at least one coder:

AIC of model with random intercept and fixed slope: 301.22

AIC of model with random intercept and random slope: 305.21
